# Cortical Trajectory Pedicle Screws for the Fixation of Traumatic Thoracolumbar Fractures

**DOI:** 10.7759/cureus.2891

**Published:** 2018-06-28

**Authors:** Jacob C Wochna, Rudy Marciano, Irina Catanescu, Joel Katz, M. Chance Spalding, Kailash Narayan

**Affiliations:** 1 Medical Student, Ohio University Heritage College of Osteopathic Medicine, Athens, USA; 2 Neurological Surgery, OhioHealth, Columbus, USA; 3 Surgery, OhioHealth, Columbus, USA; 4 Trauma Surgery, OhioHealth, Columbus, USA; 5 Neurosurgery, OhioHealth, Columbus, USA

**Keywords:** cortical bone trajectory, traumatic thoracolumbar fracture, pedicle screw, burst fracture, compression fracture, spinal fusion

## Abstract

Objective

Cortical bone trajectory pedicle screws (CBT) have a different trajectory compared to traditional pedicle screws (PS) and they may confer biomechanical advantages in some patient populations. We hypothesize that the placement of CBT in traumatic thoracolumbar fractures could be an alternative technique to the traditional utilization of PS.

Methods

Single surgeon, retrospective study was performed at a Level 1 Trauma Center from 2013 to 2017. All patients aged between 18 and 90 years with operative AO classification A, B, and C traumatic thoracolumbar fractures were included. Patients with pathological fractures, active spinal infections, or history of vertebral augmentation were excluded. Age, injury severity score (ISS), AO classification, operative time, estimated blood loss (EBL), length of stay (LOS), and presence of proximal junctional kyphosis (PJK) or construct failure were compared between CBT and PS groups. The PS group was further separated into open reduction internal fixation (ORIF) and minimally invasive spine (MIS) groups. All CBT and ORIF cases were completed via open incisions allowing arthrodesis of the involved lamina and facet joints whereas no arthrodesis was completed in the MIS patients. Choice of technique was at the attending surgeon’s discretion.

Results

The study included 71 patients, out of which 12 received CBT and 59 received PS. Of the 59 PS patients, 39 were ORIF and 20 were MIS. The average operative time was 22.9 minutes less in CBT compared to ORIF (p = 0.24). EBL was 337.50 mL for CBT, 184.33 mL for MIS, and 503.33 mL for ORIF (p = 0.01) demonstrating that MIS technique results in a significantly reduced blood loss. However, EBL was comparable for CBT versus MIS (p > 0.05). ISS was not significantly different between the three groups (p = 0.89). LOS was 4.06 days fewer for CBT patients compared to ORIF patients (p = 0.36). There was one case of construct failure as well as one case of incisional site infection in the PS group, but none were found in the CBT group. Instances of PJK complications were determined by the change in the Cobb angle over time and they were not statistically different between the three groups (p = 0.68).

Conclusions

CBT is noninferior to PS in the fixation of unstable adult traumatic thoracolumbar fractures. With the exception of EBL, CBT was not statistically different compared to MIS and ORIF. This study establishes a precedent to expand the application of this new technique and investigate with larger sample sizes.

## Introduction

Traditional pedicle screws (PS) are used as the most common fixation method for unstable traumatic thoracolumbar fractures. While cortical bone trajectory pedicle screws (CBT) have greater contact with cortical bone, it is predominately used for single level fusion in patients with lumbar degenerative pathologies and comorbidities that compromise bone quality [1-4]. We postulated that, due to increased biomechanical stability and pullout strength (POS), CBT is a noninferior treatment option [5-7]. CBT utilizes a trajectory that is medial to lateral in the transverse plane, and inferior to superior in the sagittal plane. This trajectory confers increased cortical bone purchase and a 30% increase in uniaxial POS [2, 7].

CBT is useful in patients with bone compromising comorbidities such as osteoporosis, diabetes, and tobacco abuse [2, 8]. It is thought to be a viable alternative to PS in the lower lumbar vertebra, as it allows greater contact with the cortical bone in increasingly wide pedicles [2]. This is beneficial in osteoporotic patients lacking cancellous bone mass [2]. CBT has a more medial starting point, requiring less extensive tissue dissection especially at the lower lumbar levels [9].

Recent clinical research has demonstrated that CBT is an effective alternative to PS [4, 10-11]. Multiple studies have supported CBT’s positive biomechanical properties under both physiological and nonphysiological stresses; however, others demonstrate inferiority in axial rotation and lateral bending [1, 2, 5-7, 12].

CBT's efficacy in traumatic thoracolumbar fractures has not been thoroughly investigated. This study compared the novel use of CBT in patients with unstable traumatic thoracolumbar fractures to PS in posterior spinal fusion. 

## Materials and methods

Single surgeon, retrospective review was performed at a Level 1 Trauma Center between September 1, 2013 and March 1, 2017. Institutional review board approved this study. All patients who underwent surgical fixation and posterior fusion of unstable traumatic thoracolumbar fractures were identified via trauma database query and surgeon records. Those included were between the ages of 18 and 90 years with operative AO classification A, B, or C vertebral fractures. Patients with AO subtype vertebral fractures, pathologic fractures, active infectious disease process of the spine, or history of vertebral augmentation were excluded. Patient age, injury severity score (ISS), AO classification, operative time, estimated blood loss (EBL), number of surgeries during hospital stay, length of stay (LOS), postoperative incisional infection, and presence of proximal junctional kyphosis (PJK) or construct failure were compared between CBT and PS groups.

The CBT and PS groups were compared primarily by the postoperative outcomes; presence of PJK and construct failure. Secondary focus was on the length of hospital stay, discharge disposition, total operative times, EBL, number of procedures during hospital stay, and surgical site infections.

The PS group was further separated into open reduction internal fixation (ORIF) and minimally invasive spine (MIS) groups. All CBT and ORIF cases were completed via open incisions allowing arthrodesis of the involved lamina and facet joints, whereas no arthrodesis was completed in MIS patients. The choice of technique was at the attending surgeon’s discretion.

Cobb angle was measured from the inferior endplate of the fractured vertebra to the superior endplate of the vertebra immediately above the fracture level. The change in the Cobb angle was calculated by subtracting the preoperative Cobb angle from the most recent Cobb angle based off imaging studies. Patients who presented with multiple unstable traumatic thoracolumbar fractures had their change in Cobb angle averaged over their total number of fractures for a composite value. A positive change in this value was considered PJK.

The EBL was ascertained from the surgeon’s operative note and anesthesia records. Any blood loss defined as minimal, without specifically quantifying a value, was assigned a blood loss of 100 mL. We included all surgical procedures requiring anesthesia during the hospital admission. Surgical site infections were determined by chart review, postoperative outpatient documentation, and ICD 10 code query within our trauma database registry.

Statistical analysis was performed by a trauma research biostatistician using the SAS software (Cary, NC, USA). Descriptive statistics was reported as means and standard deviations for continuous variables, and frequencies and percentages for discrete variables. Percentages were compared between/among groups using chi-square tests. Means were compared between two independent groups with two-sample t-tests; one-way analysis of variance (ANOVA) (i.e., F-tests) was used to compare three group means. Measures for which the normality assumption was questionable (i.e., number of surgeries) were compared via the Kruskal-Wallis test. A p value of <0.05 was considered statistically significant.

## Results

**Figure 1:**
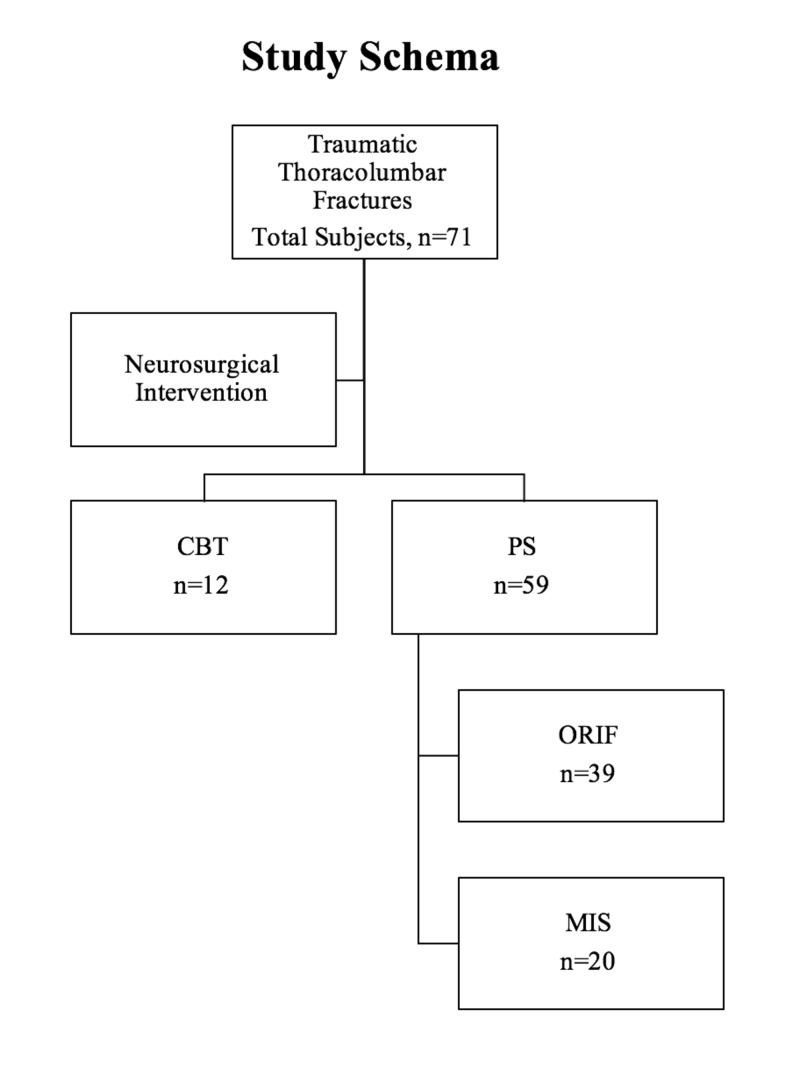
Breakdown of study population. CBT = cortical bone trajectory pedicle screw; PS = traditional pedicle screw; ORIF = open reduction, internal fixation pedicle screw; MIS = minimally invasive spine pedicle screw.

The study included 71 patients, out of which 12 received CBT and 59 received PS. Of the 59 PS patients, 39 were ORIF and 20 were MIS. Figure 1 details how patients were classified for this study.

Preoperative variables of this study are shown in Table 1; age and ISS do not have statistically significant difference between the three groups (p = 0.84 and p = 0.89, respectively). There were no statistical differences between CBT, ORIF, and MIS cohorts based on preoperative AO fracture classification (p = 0.48, see Table 2). There were no cases of C subtype fractures encountered in this study. Each fracture was classified independently because there was a subset of patients that presented with more than one unstable traumatic thoracolumbar fracture that underwent surgical fixation. This explains why the total number of fractures is greater than the number of patients found in this study.

**Table 1:** Preoperative demographic information. CBT = cortical bone trajectory pedicle screw; PS = traditional pedicle screw; ORIF = open reduction, internal fixation pedicle screw; MIS = minimally invasive spine pedicle screw.

		PS			
Procedure	CBT n = 12	Total n = 59	ORIF n = 39	MIS n = 20	p Value
Average age (years) (mean ± SD)	46.50 ± 15.13	49.24 ± 17.54	49.74 ± 16.12	48.25 ± 20.11	0.84
Average ISS (mean ± SD)	19.10 ± 13.08	18.20 ± 11.10	18.68 ± 11.70	17.19 ± 10.00	0.89

**Table 2:** Breakdown of preoperative AO fracture classifications. Total number of fractures were based off individual fractured vertebra. Cohort consisted of 71 total patients with a total of 76 vertebral fractures. CBT = cortical bone trajectory pedicle screw; PS = traditional pedicle screw; ORIF = open reduction, internal fixation pedicle screw; MIS = minimally invasive spine pedicle screw.

			PS			
Procedure		CBT n = 12	Total n = 64	ORIF n = 42	MIS n = 22	p Value
AO Classification	A1	2.00	9.00	3.00	6.00	0.48
	A2	1.00	3.00	3.00	0.00	
	A3	6.00	16.00	9.00	7.00	
	A4	2.00	18.00	13.00	5.00	
	B1	0.00	11.00	9.00	2.00	
	B2	0.00	1.00	1.00	0.00	
	B3	1.00	6.00	4.00	2.00	
	C	0.00	0.00	0.00	0.00	

Perioperative variables are displayed in Table 3; operative time, EBL, and the number of surgeries during hospital stay. The MIS cohort had a significantly less perioperative EBL when compared to ORIF (p < 0.05). On an average, EBL for CBT was 165.83 mL less than ORIF, but not statistically significantly different (337.50 mL versus 503.33 mL; p = 0.15). There were no statistically significant differences for operative time or number of surgeries during hospital stay. The average operative time was 147.67 minutes for CBT compared to 170.56 minutes in ORIF PS, resulting in a 22.9-minute difference (p = 0.24).

**Table 3:** Perioperative data. * Indicates statistical significance. CBT = cortical bone trajectory pedicle screw; PS = traditional pedicle screw; ORIF = open reduction, internal fixation pedicle screw; MIS = minimally invasive spine pedicle screw.

		PS			
Procedure	CBT n = 12	Total n = 59	ORIF n = 39	MIS n = 20	p Value
Average operative time (min) (mean ± SD)	147.67 ± 64.55	160.36 ± 70.20	170.56 ± 70.73	140.45 ± 66.37	0.24
Estimated blood loss (mL) (mean ± SD)	337.50 ± 266.39	414.72 ± 466.25	503.33 ± 502.64	184.33 ± 241.84	0.01*
Surgeries during hospital stay (no.) (mean ± SD)	1.58 ± 1.16	1.85 ± 1.37	1.97 ± 1.53	1.60 ± 0.99	0.61

**Figure 2:**
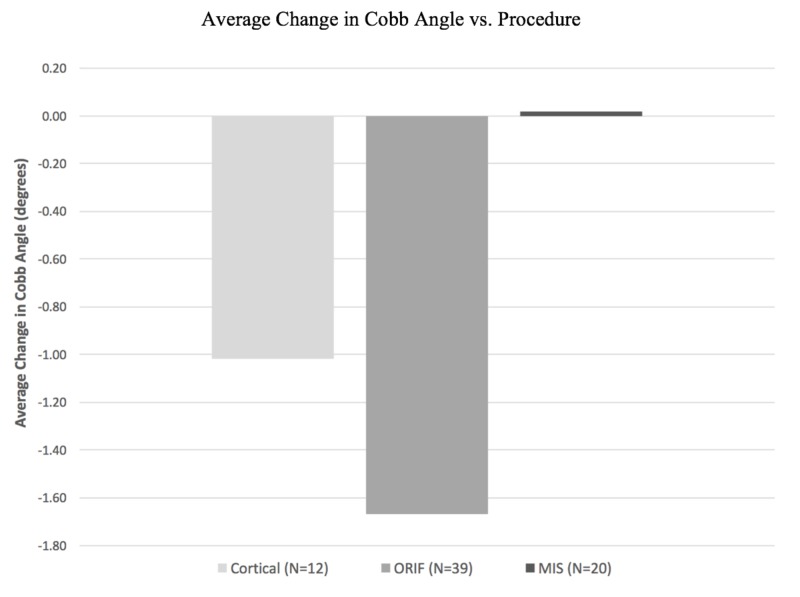
Bar graph demonstrating average change in Cobb angle of each surgical technique. Positive values indicate proximal junction kyphosis (PJK). Cortical = cortical bone trajectory pedicle screw; ORIF = open reduction, internal fixation pedicle screw; MIS = minimally invasive spine pedicle screw.

Postoperative data are displayed in Table 4; LOS, change in Cobb angle, construct failure, and discharge disposition. The average LOS was 9.83 days for CBT patients compared to 13.90 days for ORIF and 11.75 days for MIS patients (p = 0.36). The average change in Cobb angle is shown in Figure 2, which demonstrates no statistically significant difference between the three cohorts (p = 0.68). There was one case of construct failure in the PS group, but none were found in the CBT group. The ORIF group had the only documented postoperative incisional infection.

**Table 4:** Postoperative Data CBT = cortical bone trajectory pedicle screw; PS = traditional pedicle screw; ORIF = open reduction, internal fixation pedicle screw; MIS = minimally invasive spine pedicle screw.

			PS			
Procedure		CBT n = 12	Total n = 59	ORIF n = 39	MIS n = 20	p Value
Length of stay (days) (mean ± SD)		9.83 ± 5.49	13.17 ± 9.60	13.90 ± 10.00	11.75 ± 8.85	0.36
Change in Cobb angle (degrees) (mean ± SD)		-1.02 ± 6.42	-1.13 ± 6.65	-1.67 ± 7.09	0.02 ± 5.65	0.68
Construct failure (no.)		0.00	1.00	1.00	0.00	0.66
Discharge disposition	Home (no.)	5.00	26.00	20.00	6.00	
	Skilled nursing facility (no.)	4.00	16.00	10.00	6.00	
	Inpatient rehabilitation (no.)	3.00	11.00	5.00	6.00	
	Long-term acute care hospital (no.)	0.00	6.00	4.00	2.00	

## Discussion

Our results affirmed our hypothesis that the CBT surgical approach is a noninferior alternative in the fixation of adult traumatic thoracolumbar fractures. These cohorts showed similar operative times, intraoperative blood loss, LOS, and postoperative radiological and clinical outcomes.

**Figure 3:**
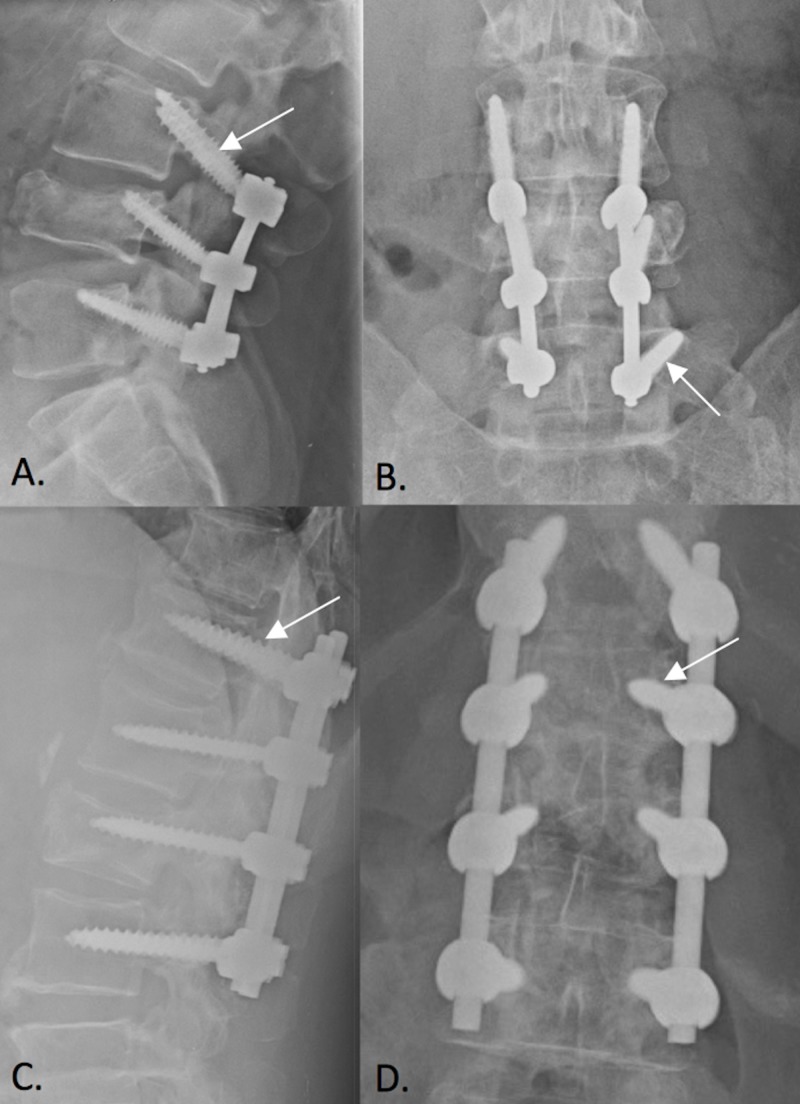
Sagittal (A) and coronal (B) cortical bone trajectory pedicle screw construct for internal stabilization of L4 burst fracture. Notice the inferior to superior trajectory in the sagittal plane and the medial to lateral trajectory in the coronal plane. Sagittal (C) and coronal (D) traditional pedicle screw construct for internal stabilization of L2 burst fracture. Notice the trajectory along the anatomic axis in the sagittal plane and the lateral to medial trajectory in the coronal plane.

This is the first study to report solely on the use of CBT for stabilization of adult traumatic thoracolumbar fractures. Although our study consisted of only 12 cases in the CBT subgroup, this is comparable to the other cohorts in the current literature. The use of CBT has been expanded to other settings, with one recent study analyzing its application in pediatric populations [4]. Sellin et al. demonstrated that the use of CBT in three pediatric patients with unstable traumatic thoracolumbar fractures was a reasonable, feasible, and safe alternative to traditional PS [4]. Figure 3 demonstrates the differences in trajectory of CBT and PS in both sagittal and coronal planes. In nontraumatic injuries, CBT has shown benefit in patients with osteoporosis due to its increased cortical bone purchase and biomechanical stability [7], but it has also shown biomechanical advantages in higher quality bone [1]. Similarly, our study discovered zero cases of construct failure or evidence of PJK with the utilization of CBT, regardless of individual comorbidity. Furthermore, there was no statistically significant difference between operative time or LOS between the three groups. Our results indicate that the MIS group attained a statistically significant reduction in EBL compared to ORIF. There was no significant difference in EBL between CBT and MIS or CBT and ORIF. Therefore, use of CBT is a plausible alternative to PS.

**Figure 4:**
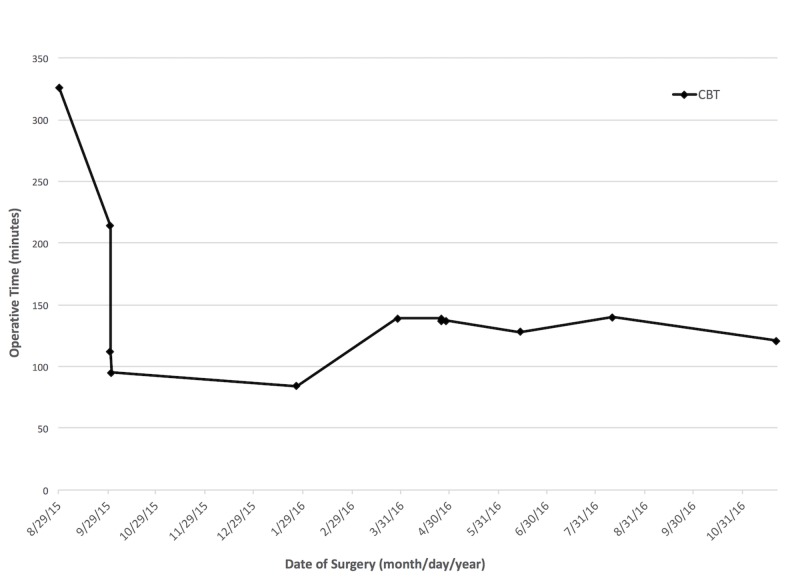
Chronological progression of operative times with cortical bone trajectory pedicle screw technique, from first to last case, demonstrates the reduction in operative time associated with increased familiarity. CBT = cortical bone trajectory pedicle screw.

CBT was not a regularly performed procedure at our institution, which led to a learning curve, as demonstrated by the chronological decrease in operative times shown in Figure 4. While not achieving statistical significance, the CBT technique was, on average, faster than ORIF. As technical familiarity increases, we postulate that continued studies of this technique will reveal improved operative times. 

Due to the medial starting point and decreased paraspinal muscle tissue dissection and retraction [9], previous studies have shown CBT to have decreased intraoperative EBL [10-11]. This result was not reproduced in our study; however, the MIS subgroup had a significantly lower EBL compared to ORIF (p < 0.05). The decreased tissue dissection and retraction are also thought to lead to decreased postoperative pain and reduced risk of surgical site infection [10]; of which we only saw one, in the PS ORIF category. Narcotic use and further recording of surgical site infections will be variables of future study at our institution.

A major limitation to this study is the greater distribution of AO Type B fractures in the ORIF group, signifying a nonrandomized distribution. The primary surgeon more frequently utilized ORIF, rather than MIS or CBT, on the more severe Type B fractures given the increased ability to obtain extensive posterolateral arthrodesis conferred by the wider exposure. Future studies should be randomized.

Other areas of potential limitation to our study include small sample size, long-term follow up, and fracture level delineation. Although our CBT population volume was comparable to similar studies in the literature, a power analysis was performed, which revealed, with 80% certainty, that we required an additional 244 patients to make it a sufficiently powered study. As the CBT technique continues to be utilized at our institution in adult trauma patients, future studies will focus on increasing the sample size and long-term follow up examining PJK and construct failure. Long-term follow up at regular intervals is essential in identifying the comparative effectiveness of CBT versus PS; unfortunately, due to lack of consistent follow up, we do not have data on the long-term outcomes from patients in this study. Additionally, this clinical study did not assess the efficacy of PS versus CBT based on vertebral level. Sansur et al. studied the varied peak load of failure at different vertebral levels for both CBT and PS. Findings showed that there was a marked increase in the mean load of failure at lower vertebral segments with CBT, and found the opposite trend for PS [2]. There is a value in future clinical studies, which compares PS versus CBT based on vertebral level because of the dynamic changes in cancellous and cortical bone, transverse pedicle angle, and pedicle volume at different levels along the spine. In such a study, we may be able to determine if there is an optimal application of CBT based on the vertebral level of traumatic thoracolumbar fracture.

Future studies should analyze the strengths and weaknesses of the construct design comparatively for CBT versus PS. CBT is superior in flexion and extension, while PS is superior in side bending and rotation [6]. Many of these outcomes will be measured in the prospective, randomized controlled trial that is underway comparing CBT to PS [3]. However, it is not yet known whether increased screw contact in denser cortical bone with CBT or increased resistance in side bending and rotational physiologic stresses with PS is more important in construct stability. Furthermore, a cross-link can be used with CBT constructs to increase rotational stability, however, this was not done in our series.

The CBT has been reported as beneficial in a multitude of different patient populations due to its medial starting point and biomechanical advantages [1-2, 4, 10]. Current literature has described CBT as having decreased EBL, LOS, postoperative pain, and a low complication profile [10-11]. Our study found that CBT is a safe, plausible alternative modality to traditional PS in the fixation of adult traumatic thoracolumbar fractures.

## Conclusions

This study demonstrates that CBT is a potential alternative technique in adult traumatic thoracolumbar fractures. When compared to PS ORIF and MIS techniques, intraoperative, postoperative, radiological, and clinical outcomes were comparable in our patient population. The MIS techniques resulted in lesser blood loss than CBT and ORIF. This was an initial descriptive study of a known technique used for an under-reported indication, with the results demonstrating that CBT is a safe alternative that warrants further investigation.
